# Cross-reaction of POC-CCA urine test for detection of *Schistosoma mekongi* in Lao PDR: a cross-sectional study

**DOI:** 10.1186/s40249-020-00733-z

**Published:** 2020-08-12

**Authors:** Anousin Homsana, Peter Odermatt, Phonesavanh Southisavath, Aya Yajima, Somphou Sayasone

**Affiliations:** 1 Lao Tropical and Public Health Institute, Ministry of Health, Vientiane Capital, Lao People’s Democratic Republic; 2 Department of Epidemiology and Public Health, Swiss Tropical and Public Health Institute, P.O. Box, CH-4002 Basel, Switzerland; 3 University of Basel, P.O. Box, CH-4003 Basel, Switzerland; 4 Department of Radiology, Mahosot Hospital, Ministry of Health, Vientiane Capital, Lao People’s Democratic Republic; 5 World Health Organization, Western Pacific Regional Office, Manila, Philippines

**Keywords:** Point-of-care circulating cathodic antigen, Lao People’s Democratic Republic, Kato-Katz, *Schistosoma mekongi*, *Opisthorchis viverrini*, Soil-transmitted helminth

## Abstract

**Background:**

The point-of-care circulating cathodic antigen (POC-CCA) test is increasingly used as a rapid diagnostic method for *Schistosoma mansoni* infection. The test has good sensitivity, although false positive results have been reported among pregnant women and patients with urine infections and hematuria. We validated the POC-CCA test’s ability to diagnose *Schistosoma mekongi* infection in Lao People’s Democratic Republic (Lao PDR), where *S. mekongi* is endemic. Of particular interest was the test’s specificity and possible cross-reactivity with other helminth infections*.*

**Methods:**

We conducted a cross-sectional study of children and adults in the provinces of Champasack (*Schistosoma mekongi* and *Opisthorchis viverrini* endemic), Savannakhet (*O. viverrini* endemic) and Luang Prabang (soil-transmitted helminths endemic) between October 2018 and April 2019**.** POC-CCA and urine dipstick tests were administered to all study participants, while an additional pregnancy test was offered to women. Two stool samples were collected from participants and examined with a Kato-Katz test (two smears per stool). Logistic regression was used to associate potential confounding factors (predictors) with POC-CCA test results (outcome).

**Results:**

In *S. mekongi*-endemic Champasack, 11.5% (*n* = 366) and 0.5% (*n* = 2) of study participants had positive POC-CCA and Kato-Katz test results, respectively. Only one of the two Kato-Katz positive patients was also POC-CCA positive. In Champasack and Luang Prabang, where *S. mekongi* is not endemic, the POC-CCA test yielded (presumably) false positive results for 6.0% (*n* = 22) and 2.5% (*n* = 9) of study participants, respectively, while all of the Kato-Katz tests were negative. POC-CCA positive test results were significantly associated with *O. viverrini* infection (1.69, 95% confidence interval (*CI*): 1.02–2.77, *P* = 0.042), increased leukocytes (adjusted Odds Ratio *(*a*OR*) = 1.58, 95% *CI*: 1.15–2.17, *P* = 0.005) and hematuria (a*OR* = 1.50, 95% *CI*: 1.07–2.10, *P* = 0.019) if the observed trace was counted as a positive test result. Two pregnant women from Champasack province had POC-CCA positive tests.

**Conclusions:**

We observed a cross-reaction between the POC-CCA test and *O. viverrini* infection. To some extent, we can confirm previous observations asserting that POC-CCA provides false positive results among patients with urinary tract infections and hematuria. In *S. mekongi*-endemic areas, POC-CCA can be applied cautiously for surveillance purposes, keeping in mind the considerable risk of false positive results and its unknown sensitivity.

## Introduction

The schistosomiasis, caused by the *Schistosoma mekongi* blood fluke, affects communities in the Mekong River Basin, particularly those in southern Lao People’s Democratic Republic (Lao PDR) and in northern Cambodia [1]. The parasite was first discovered in 1957, in a Lao patient hospitalized in Paris, France [2]. In the 1980s, the public health problem posed by the infection was recognized in two districts (Khong and Mounlapamok) in Champasack province, Lao PDR. The first round of community-based chemotherapy intervention was implemented from 1989 to 1999; a second round started in 2007, and continues to this day [3, 4]. As a result, the prevalence of *S. mekongi* infection and heavy infection intensity in the two districts has fallen to less than 5% and 1%, respectively [5].

In 2016, the Ministry of Health (MOH) in Lao PDR adopted a national action plan for eliminating schistosomiasis in the two endemic districts, with the goals of eliminating schistosomiasis as a public health problem by 2018, interrupting transmission by 2025 and verifying elimination by 2030 [5]. To this end, a surveillance system was set up to monitor the progress of intervention. It includes annually monitoring *S. mekongi* infection via village sentinel sites and rotating village spot checks. Currently, the gold standard for detecting intestinal schistosomiasis, recommended by the World Health Organization (WHO), is the Kato Katz (KK) test, which detects eggs in stool by staining a sieved fecal sample and examining it under a microscope [5]. As such, the Lao PDR national schistosomiasis elimination program continues to rely on this method to monitor *S. mekongi* infection. However, it is widely recognized that the Kato-Katz technique is not particularly sensitive to light-intensity infections; hence, more sensitive diagnostic tools are urgently required to continue monitoring the impacts of interventions in low-prevalence settings and, ultimately, to verify the absence of schistosomiasis transmission [6].

Recently, more sensitive diagnostic tools have been developed to detect circulating antigens of schistosomes in the host’s blood and urine [7, 8]. One such tool is the rapid point-of-care test to detect circulating cathodic *S. mansoni* antigens (POC-CCA) in urine [7]. In a study of asymptomatic Eritrean refugees from *S. mansoni*-endemic areas, POC-CCA test in urine showed high sensitivity (91.3%) [9]. In 2015, WHO recommended the use of POC-CCA for mapping *S. mansoni* endemicity in affected countries [10].

The *S. mansoni* POC-CCA test was applied in *S. mekongi-*endemic areas of Lao PDR (Khong district, Champasack province) and Cambodia (Kratié province) in the year 2017 [11]. The study found that while the KK test detected *S. mekongi* eggs in 6.4% of the 377 study participants using three samples per participant, 21.0% of the study participants tested positive with POC-CCA. If trace observations were also considered positive, 53.9% of study participants would have been considered infected [11]. However, when urine POC-CCA and KK results were compared to the overall composite assessment reference, POC-CCA sensitivity was only 24.1% but still almost two times higher than that of the KK method (13.6%) [11].

A further study to validate the POC-CCA urine test in asymptomatic Eritrean refugees from *S. mansoni*-affected areas confirmed the high sensitivity of 90%, but showed a relatively low specificity of 73.6% [12]. A follow-up study of individuals who had never visited a *Schistosoma*-endemic area (residents of Switzerland), indicated a considerable amount of false positive (low specificity) cases among healthy adults (80% specificity), infants under three years (83% specificity), pregnant women (80% specificity), patients with urinary tract infections (73% specificity), patients with hematuria (93% specificity) and patients with bladder malignancies (80% specificity) [13]. This raised a concern about the use of POC-CCA in Southeast Asian settings where many other parasitic diseases are co-endemic, especially those caused by trematode species, which might cross-react with POC-CCA.

The concerns regarding the test’s low specificity and potential cross-reactions with other highly prevalent and related trematode species, particularly *Opisthorchis viverrini* and different minute intestinal fluke (MIF) species in Lao PDR, warranted further validation of the test. We assessed the performance of the *S. mansoni* POC-CCA urine test in *S. mekongi*-endemic areas and non-endemic areas of Lao PDR, in terms of specificity and potential cross-reaction with other helminth infections and pathologies.

## Materials and methods

### Study area and population

**Fig. 1:**
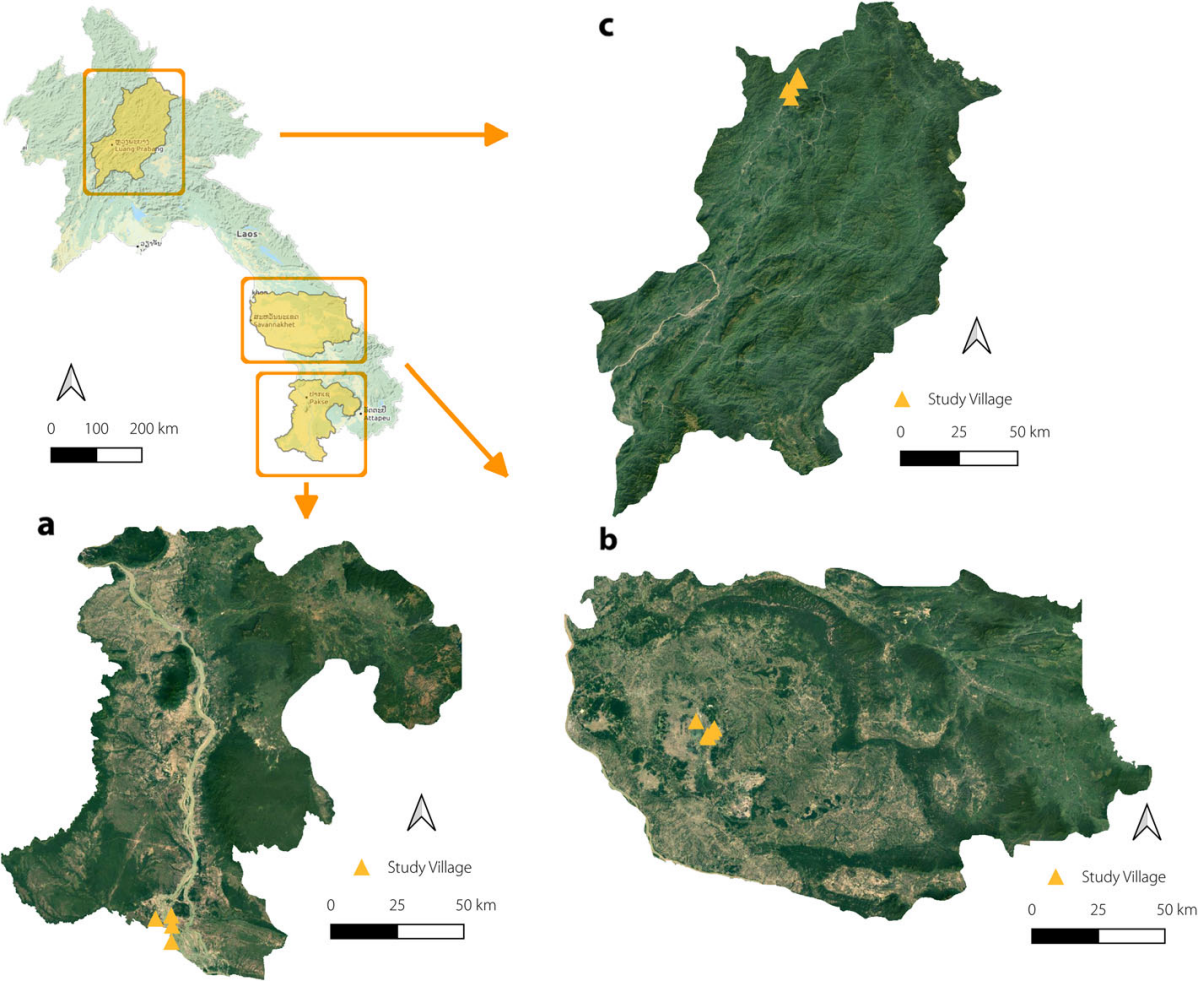
Map of Lao PDR and study provinces and villages in **a**) Champasack, **b**) Savannakhet and **c**) Luang Prabang provinces. (sources: OpenStreetMap contributors, 2015; Map data©2015 google)

This study was conducted in three different helminth-endemic settings in Lao PDR, specifically one district per province in Champasack, Savannakhet and Luang Prabang. Khong district, in Champasack province, is located in the southern end of the country (geographical coordinates: 14.094281, 105.792992). Figure 1 shows a map of the study location. It borders Cambodia and has a population of 80 000 [14]. *S. mekongi*, *O. viverrini* and soil-transmitted helminth (STH) are known to be endemic in this district [15]. Champhone district, in Savannakhet province, is situated in the central part of the country (geographical coordinates: 16.528993, 105.234703) and is populated by some 100 000 people [14]. In this area for *O. viverrini*, intestinal trematodes and STH [16] but not for *S. mekongi*. Namback District, in Luang Prabang Province, is in the northern part of Lao PDR (geographical coordinates: 20.630490, 102.467147) and has a total of 68 863 people [14]. Namback is highly endemic for STH, but not for *S. mekongi* nor *O. viverrini* [17].

**Fig. 2:**
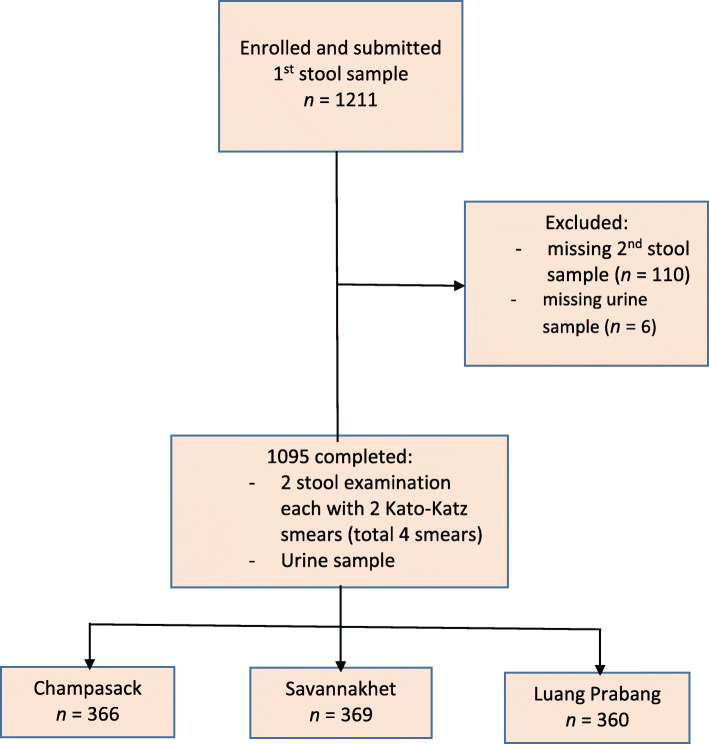
Study enrollment

### Study design

For this cross-sectional study, we used purposive sampling. Five study villages and five primary schools were selected in each district. In each village selected, about 40–60 adults aged 35 years and older were invited to participate. The selection process went as follows: (i) village demographic information was obtained from the village register, available at the district health offices; (ii) study households in each village were contacted with the help of the village leaders, while the heads of primary schools provided student lists; and (iii) all adult members of the selected household, aged 35 years and older and at home on the days of study were invited to participate. Primary school students were also invited to participate. From each primary school, 30–40 students from grades 3 to 5 were included in the study.

The study was carried out between October 2018 and April 2019 (from October to December 2018 in Champasack; between January and February 2019 in Savannakhet; and between March and April 2019 in Luang Prabang).

### Field and laboratory procedures

#### Stool examination

Each study participant was asked to provide two stool samples on two consecutive days for parasitological analysis using the KK method. Two KK smears were prepared from each stool sample (total four KK smears per person). The smears were allowed to clear for 30 to 60 min prior to examination under a light microscope, conducted by experienced microscopists. All helminth eggs were counted and recorded for each species separately.

#### POC-CCA strip

A 20-centiliter urine sample was collected from each study participant and examined immediately. The POC-CCA strip (Schistosome POC-CCA Test, ICT Diagnostics, Cape Town South Africa) required 100 μl urine and the test was read after 20 min*.* The results were interpreted and recorded according to the manufacturer’s instructions on test band intensity, i.e. positive when the test yields a pink band, trace positive when the test band is barely visible, and negative when no band appears [18].

#### Urine analysis

Additional urine analysis was performed using a dipstick (Combur 10, Roche, Germany). Blood in the urine (hematuria), leukocyte and nitrite, as well as protein (proteinuria) are among the 10 signs suggestive of a urinary tract infection [19, 20]. The test strip was dipped in the urine sample and the result was read after 1 min, according to the manufacturer’s instructions. The intensity of the reaction zone was compared to the color label provided on the dipstick box. Categorical numbers correspond to the substance concentration in the urine sample, thereby providing cut-off values for positive or negative results. For example, leucocytes is classified as negative (< 10 leu/μl), 1+ (~ 10–25 leu/μl), 2+ (~ 75–500 leu/μl), or 3+ (~ 500 leu/μl); nitrite is negative (detection limit < 0.5 mg/L) or positive; blood erythrocyte is negative, 1+ (≥ 10–25 ery/μl), 2+ (≥ 25–50 ery/μl), 3+ (≥ 50–250 ery/μl) or 4+ (≥ 250 ery/μl)); while proteinuria can be negative (< 0.1 g/L), 1+ (0.3 g/L), 2+ (1 g/L), or 3+ (5 g/L)) [21].

A pregnancy test (One Step Pregnancy Test, AI Diagnostic, Shandong, China) was performed with samples from all female participants between 12 and 45 years of age.

### Data management and statistical analysis

Data were collected and stored using electronic tablets. The questionnaires and forms had been developed in the Commcare server (www.commcarehq.org). Commcare ODK, version 3.4, was installed on the tablets for field data collection. The completed forms were converted to Microsoft Excel version 16 (Microsoft Cooperation, Washington, USA) files and checked for completeness and consistency.

Only individuals with complete datasets were included in the final analysis. All statistical analysis was performed using STATA (version 16, Stata Cooperation, Texas, USA).

Descriptive statistics (frequency, proportions and means) were applied to describe the study participants’ socio-demographic characteristics, infection status and infection intensity. The results of the POC-CCA urine test for the detection of *S. mekongi* was classified into one of two scenarios: (i) a reliable scenario, where a trace observation was considered as a negative result; and (ii) a doubtful scenario, where a trace observation was considered as a positive result. A bivariate logistic regression model was used to associate the results of the POC-CCA urine test (outcome) with covariates, such as study locations, demographic information, helminth infections, and urine analysis results (the presence of leukocytes and/or nitrites were combined into one variable indicating urinary tract infection). The crude odds ratio (c*OR*) and 95% confidence intervals (95% *CI*) were calculated. Variables with a significance level less than 20% were included in the multivariate logistic regression analysis. The adjusted odds ratio (a*OR*) and 95% *CI* were calculated. All variables with *P*-values less than 0.05 were considered statistically significant.

## Results

### Enrollment of study participants

Of the household members and school children who had been informed of our study, 1211 individuals were enrolled and submitted stool samples. In total, 1095 participants completed all requirements and were included in the final analysis; 366, 369, and 360 participants came from Champasack, Savannakhet and Luang Prabang, respectively (Fig. 2).

### Socio-demographic characteristics of the study participants

The socio-demographic characteristics of the study participants from all three study sites were collected, as shown in Table 1. Slightly more than half of the participants were females (54.4%). The study population consisted of children below 13 years of age (33.8%) and adults aged 35 years or older (66.2%). The proportion of adult participants was slightly higher in Champasack (56.8%) and in Savannakhet (56.4%). However, in Luang Prabang, only 14.5% were children. The majority of participants (67.1%) belonged to the Lao-Tai ethnic group. Most participants had finished primary school (70.9%) and were farmers (62.2%).

**Table 1:** Socio-demographic characteristics of the study participants

Indicators	Champasack, *n* = 366 % (***n***)	Savannakhet, *n*= 369 ‚% (***n***)	Luang Prabang, *n* = 360 ‚% (***n***)	Total, *n* = 1059 ‚% (***n***)
**Gender**				
Male	45.9 (168)	42.5 (157)	48.3 (174)	45.6 (499)
Female	54.1 (198)	57.5 (212)	51.7 (186)	54.4 (596)
**Age in year**				
≤ 13 Mean (min − max)	43.2 (158) 9.5 (8–13)	43.6 (161) 9.5 (7–13)	14.2 (51) 10.3 (8–13)	33.8 (307) 9.4 (7–13)
≥ 35	56.8 (208)	56.4 (208)	85.8 (309)	66.2 (725)
Mean (min − max)	50.1 (35–77)	52.7 (35–83)	47.2 (35–60)	49.6 (35–83)
**Ethnicity**				
Lao-Tai	100 (366)	100 (369)	0 (0)	67.1 (735)
Minority (Lao-Theung)	0 (0)	0 (0)	100 (360)	32.9 (360)
**Education**				
Illiterate	5.7 (21)	10.7 (38)	31.9 (115)	15.9 (174)
Primary	78.7 (288)	70.7 (261)	63.3 (228)	70.9 (777)
Secondary	15.6 (57)	16.3 (60)	4.7 (17)	12.2 (134)
University	0 (0)	2.7 (10)	0 (0)	0.9 (10)
**Profession**				
Farmer	53.5 (196)	53.7 (198)	85.8 (309)	64.2 (703)
Student	43.2 (158)	43.6 (161)	14.2 (51)	33.8 (307)
Fisherman	3.3 (12)	0 (0)	0 (0)	1.1 (12)
Teacher	0 (0)	2.7 (10)	0 (0)	0.9 (10)

### Helminth infection diagnosed by Kato-Katz test

The results of the KK stool examination were summarized in Table 2. Only two study participants (0.5%) were found to be infected with *S. mekongi*. Both were from *S. mekongi* endemic villages in Champasack. In the two other provinces (Savannakhet and Luang Prabang), no *S. mekongi* infection was detected. The prevalence of *O. viverrini* infection was very high in Champasack (62.9%) and in Savannakhet (77.2%), and low in Luang Prabang (12.2%). The prevalence of hookworm infection was high in all three study sites: 40.4% in Champasack; 68.6% in Savannakhet, and 86.4% in Luang Prabang. STH infections, namely *T. trichiura* and *A. lumbricoides,* were rarely diagnosed in Champasack (0.5 and 0.2%, respectively) and Savannakhet (1.4 and 0.3%), but were highly prevalent in Luang Prabang (31.7 and 12.5%).

**Table 2:** Results of Kato-Katz stool examination: Prevalence and intensity of helminth infections

Parasites ^**a**^	Champasack, ***n*** = 366		Savannakhet, ***n*** = 369		Luang Prabang, ***n*** = 360		Total, ***n*** = 1095	
	% (*n*)	Mean EPG (Max−Min)	% (*n*)	Mean EPG (Max−Min)	% (*n*)	Mean EPG (Max−Min)	% (*n*)	Mean EPG (Max−Min)
*Schistosoma mekongi*	0.5 (2)	12 (12)	0 (0)	0 (0)	0 (0)	0 (0)	0.2 (2)	12 (12)
*Opisthorchis viverrini*	62.8 (230)	2352 (6–41 700)	77.2 (285)	1343 (6–29 538)	12.2 (44)	81 (6–1152)	51.1 (559)	1659 (6–41 700)
Hookworm	40.4 (148)	300 (6–5472)	68.6 (253)	340 (6–6306)	86.4 (311)	1207 (6–14 004)	65.0 (712)	710 (6–14 004)
*Trichuris trichiura*	0.5 (2)	18 (12–24)	1.4 (5)	72 (24–108)	31.7 (114)	50 (6–306)	11.1 (121)	50 (6–306)
*Ascaris lumbricoides*	0.5 (2)	228 (78–378)	0.3 (1)	12 (12)	12.5 (45)	5429 (24–71 970)	4.4 ()	5100 (12–71 970)

^a^ Overall prevalence of *Enterobius vermicularis* and *Taenia* spp. was 1.8 and 2.0%, respectively. EPG: Eggs per gram

### POC-CCA test performance

Overall, 73 participants (6.7%) had positive POC-CCA tests (Table 3). The highest positivity rate was observed in the *S. mekongi-*endemic Champasack province (11.5%), while Savannakhet and Luang Prabang had rates of 6.0 and 2.5%, respectively. When trace observations were considered as positive, the positivity rate almost tripled, with a high of 39.1% in Champasack. Even in Savannakhet and Luang Prabang, where *Schistosoma* is not endemic, considerably high positivity rates were observed: 14.6 and 7.8%, respectively.

**Table 3:** POC-CCA test in urine: Performance in diagnosing *Schistosoma mekongi* in three provinces in Lao PDR (*n* = 1095)

Indicators	Champasack, *n *= 366 % (***n***)	Savannakhet, *n* = 36 % (*n*)	Luang Prabang,* n *= 360 % (*n*)	Total, *n* = 1059 % (***n***)
**POC-CCA analysis**				
Positive	11.5 (42)	6.0 (22)	2.5 (9)	6.7 (73)
Trace	27.6 (101)	8.7 (32)	5.3 (19)	13.9 (152)
Negative	60.9 (223)	85.4 (315)	92.2 (332)	79.5 (870)
**Trace considered as negative**				
Positive	11.5 (42)	6.0 (22)	2.5 (9)	6.7 (73)
Negative	88.5 (324)	94.0 (347)	97.5 (351)	93.3 (1022)
**Trace considered as positive**				
Positive	39.1 (143)	14.6 (54)	7.8 (28)	20.5 (225)
Negative	60.9 (223)	85.4 (315)	92.2 (332)	79.5 (870)

*POC-CCA* Point-of-care circulating cathodic Schistosoma mansoni antigen Lao, *PDR* Lao People’s Democratic Republic

### Urine analysis

The results of the urine analysis to detect urinary tract abnormalities were summarized in Table 4. Overall, one-third (30.9%) of the urine samples contained leucocytes. A few participants (3.0%) tested positive for nitrite. Erythrocytes were detected in about one quarter of the samples (24.5%), while proteinuria was found in 12.5% of all urine samples. Eight participants (aged ≥ 12 years) (0.7%) had a positive pregnancy test result.

**Table 4:** Urine analysis to detect urinary tract abnormalities among all study participants

Parameters	Champasack, *n* = 366 % ***(n***)	Savannakhet, *n* = 369 % (***n***)	Luang Prabang, *n* = 360 % (***n***)	Total, *n* = 1095 % (***n***)
**Urinalysis, % (*n*)**				
Leukocyte (1+, 2+ and 3+)	33.6 (123)	32.8 (121)	26.1 (94)	30.9 (338)
Nitrite (positive)	3.6 (13)	4.1(15)	1.4 (5)	3.0 (33)
Erythrocyte (1+, 2+, 3+ and 4+)	23.0 (84)	22.8 (84)	27.8 (100)	24.5 (268)
Protein (1+, 2+ and 3+)	5.7 (21)	8.7 (32)	23.3 (84)	12.5 (137)
**Pregnancy test**				
Positive	1.1 (4)	0.3 (1)	0.8 (3)	0.7 (8)

### Association between POC-CCA results and other factors

The results of the bivariate association between POC-CCA urine test outcomes and various covariates (additional file 1). In the first model, an observed trace counted as a negative result, and in the second model, it counted as a positive result. The strongest associations were observed between the POC-CCA trace-positive outcomes and helminth infections. For example, the association with *O. viverrini* infection was statistically significant, with an *OR* of 1.65 (95% *CI*: 1.22–2.23, *P* = 0.001). The presence of leukocytes and/or nitrite in the urine showed significant positive associations with both trace-negative (*OR* = 1.78, 95% *CI*: 1.10–2.89, *P* = 0018) and trace-positive (*OR* = 1.79, 95% *CI*: 1.32–2.42, *P* = 0.001) models. Likewise, the presence of hematuria showed significant positive associations with both trace-negative (*OR* = 1.67, 95% *CI*: 1.01–2.76, *P* = 0.046) and trace-positive (*OR* = 162, 95% *CI*: 1.17–2.23, *P* = 0.003) models. None of the regression models associated POC-CCA test outcomes with *S. mekongi* infection. One of the two individuals who tested positive for *S. mekongi* eggs with the KK technique also had a clear positive POC-CCA test, while the other KK-positive individual had a trace-positive POC-CCA result. Of the eight participants who tested positive for pregnancy, one had a clear positive POC-CCA test, while one other had a trace-positive POC-CCA result. Both of these pregnant women originated from Champasack province.

The results of the multivariate analysis of POC-CCA urine test outcomes with all covariates that had a significance level of less than 20% in the bivariate analysis are shown in Table 5. Again, the analysis was performed for each of the two models, where trace observations were considered as a negative or positive test outcome, respectively. In both models, *O. viverrini* and hookworm infections were significantly associated with the POC-CCA test outcomes. Stronger associations were detected in the trace-positive model, particularly between POC-CCA positivity and *O. viverrini* infection (a*OR* = 1.75, 95% *CI*: 1.29–2.40, *P* < 0.001) and between POC-CCA negativity and hookworm infection (a*OR* = 0.51, 95% *CI*: 0.38–0.70, *P* < 0001). Hematuria (a*OR* = 1.50, 95% *CI*: 1.07–2.10, *P* = 0.019) and leucocytes/nitrite in urine (a*OR* = 1.58, 95% *CI*: 1.15–2.17, *P* = 0.005) were also positively and significantly associated with POC-CCA test outcomes in the trace-positive model; proteinuria was associated with POC-CCA positivity at the 6% level. No other covariate had a statistically significant association with POC-CCA test outcomes.

**Table 5:** Multivariate analysis of associations between POC-CCA outcomes and its covariates among study participants in three provinces in Lao PDR (*n* = 1095)

Indicators	Trace as negative		Trace as positive	
	a***OR*** (95% ***CI***)	***P-***value	a***OR*** (95% ***CI***)	***P-***value
**Helminth infections**				
*O. viverrini*	1.67 (1.02–2.77)	0.042	1.75 (1.29–2.40)	< 0.001
Hookworm	0.52 (0.32–0.85)	0.010	0.51 (0.38–0.70)	< 0.001
**Urine analysis**				
Leukocyte/Nitrite	1.54 (0.93–2.54)	0.090	1.58 (1.15–2.17)	0.005
Hematuria	1.52 (0.90–2.57)	0.119	1.50 (1.07–2.10)	0.019
Proteinuria	1.63 (0.83–3.17)	0.153	1.54 (0.99–2.40)	0.056

a*OR*: Adjusted odds ratio; *CI*: Confidence interval

## Discussion

The POC-CCA test in urine is one of the most efficient tests for diagnosing an active *S. mansoni* infection, particularly in field settings [7]. It detects circulating cathodic *S. mansoni* antigens in the urine of infected patients [22]. POC-CCA has been extensively evaluated in areas highly-endemic for *S. mansoni*. The results of a study performed in five African countries document an overall test sensitivity of 86% [23], demonstrating that the POC-CCA test has a higher sensitivity compared to the KK technique. Good POC-CCA test sensitivity was also observed in a Swiss study of asymptomatic refugees from a country highly endemic for *S. mansoni* [9]; the results showed a sensitivity of 91%. Moreover, in areas of Brazil with low *S. mansoni* endemicity, POC-CCA also showed good sensitivity when trace observations were considered as positive results [24].

Still, evidence from other studies give rise to concerns about POC-CCA test specificity and possible interactions with other conditions, such as urinary tract infection, hematuria [13], and pregnancy [25]. Our previous study of *S. mekongi*-endemic areas in Lao PDR and Cambodia showed an exceedingly high positivity rate with POC-CCA testing, suggesting that other, closely related helminth infections, such as *O. viverrini*, might lead to positive POC-CCA test results [11].

Taking into account these concerns, we aimed to further clarify if the *S. mansoni*-based POC-CCA test in urine is suitable for diagnosing an active *S. mekongi* infection. In particular, we wanted to clarify the test’s specificity in the context of Lao PDR. To do this, we performed a cross-sectional study of children and adults in three provinces in Lao PDR; only one province, Champasack, was known to be endemic for *S. mekongi*.

Overall, we found POC-CCA test positivity rates of 6.7 and 20.5% if trace observations were considered as negative or positive, respectively. Among the three provinces, *S. mekongi-*endemic Champasack had the highest positivity rate of 11.5%. However, considerable positivity rates were also observed in Savannakhet and Luang Prabang, 6.0 and 2.5%, respectively. As neither province is endemic for *S. mekongi*, the results indicate a high rate of false-positive POC-CCA test results. When POC-CCA trace observations were considered as positive results, the positivity rates tripled.

We found that POC-CCA test results were associated with other helminth infections. In particular, POC-CCA test positivity was positively and significantly associated with *O. viverrini* infection. This observed association is evidence of a likely cross-reaction since *O. viverrini* is a trematode closely related to the *Schistosoma* species. It is a major public health problem in Lao PDR [15, 16], and highly prevalent in the central and southern regions of the country, including Champasack and Savannakhet provinces. Further, POC-CCA test results were also associated with hookworm infection when trace observations were considered as positive results; individuals with positive POC-CCA tests had a lower risk of hookworm infection compared to those with negative POC-CCA tests. This observation is best explained by the geographical concurrence of hookworm infection and not by cross-reactivity*.* Thus, to confirm the cross-reactivity of POC-CCA test and other helminth infections, an alternative approach can be used for future studies such as pooling of samples with known combinations of parasites or a co-examination with the more specific polymerase chain reaction method.

Previous studies found that POC-CCA tests yield false positive results in patients with urine infections, hematuria, and proteinuria [13]. In our study, we found only partial evidence of these associations. Although these conditions were associated with POC-CCA test results in the univariate analysis, they were only associated in the multivariable analysis when trace observations were considered as positive. This finding might be explained by the fact that, unlike the previous studies, our study was conducted in a community setting and not in a clinic.

Of the eight pregnant women in our study, one tested positive with POC-CCA and another tested trace positive. As these two women originated from Champasack, our study does not provide any evidence that pregnancy leads to false positive POC-CCA results. However, the cross-reaction between POC-CCA and pregnancy has been observed in two other studies. One 2016 study found that two pregnant women from Switzerland, with no history of *S. mansoni* infection, tested positive with POC-CCA [25]. Similarly, a 2019 study from Switzerland [13] showed that 3 out of 15 pregnant women residing in Switzerland tested positive for *S. mansoni* infection with POC-CCA.

The low number of *S. mekongi*-confirmed patients in our study group makes it difficult to provide a meaningful sensitivity estimate for POC-CCA testing. Over the last decade, annual mass drug administration combined with community awareness programs have reduced *S. mekongi* infection rates to a low level [26]. Our study found only two patients with a confirmed infection, compared to our previous study in 2017 [11], where 6.4% (*n* = 24) of study participants tested positive with the KK technique. In future studies, we will need to take into account further the low infection rates by examining an increased number of stool samples and smears per stool in order to further increase the sensitivity of KK [27].

## Conclusions

Using POC-CCA tests in areas that are not endemic for *S. mekongi* leads to many false-positive results in Lao PDR, due to cross-reactivity with *O. viverrini* infection. In the *S. mekongi*-endemic areas of Lao PDR and Cambodia, we recommend caution when using POC-CCA testing; positive cases may require more rigorous parasitological testing to confirm the infection.

## Supplementary information

**Additional file 1. **Bivariate analysis of associations between POC-CCA outcomes and covariates among study participants living in three provinces in Lao PDR (*n* = 1095).

## Data Availability

Data is available from the corresponding author upon reasonable request.

## Abbreviations

a*OR*: Adjusted *Odds Ratio*
*CI*: Confidence interval
*OR*: crude Odds Ratio
EPG: Eggs per gram
KK: Kato-Katz
Lao PDR: Lao People’s Democratic Republic
MDA: Mass drug administration
*O. viverrini*: *Opisthorchis viverrini*
POC-CCA: Point-of-care circulating cathodic *Schistosoma mansoni* antigen
PZQ: Praziquantel
STH: Soil-transmitted helminth
*S. mekongi*: *Schistosoma mekongi*
