# Supplementary material for: Cross-reaction of POC-CCA urine test for detection of Schistosoma mekongi in Lao PDR: a cross-sectional study
Source: Infect Dis Poverty. 2020 Aug 12;9:114. doi: 10.1186/s40249-020-00733-z (PMC7424653; doi:10.1186/s40249-020-00733-z)
Supplement: Supplementary file 1 — Additional file 1. Bivariate analysis of associations between POC-CCA outcomes and covariates among study participants living in three provinces in Lao PDR (n = 1095). [file 40249_2020_733_MOESM1_ESM.docx]

| **Indicators** | **Trace as negative** | |  | **Trace as positive** | |
| --- | --- | --- | --- | --- | --- |
|  | **c*OR* (95% *CI*)** | ***P-*value** |  | **c*OR* (95% *CI*)** | ***P-*value** |
| **Demographic** |  |  |  |  |  |
| **Sex** |  |  |  |  |  |
| Female | 1.00 |  |  | 1.00 |  |
| Male | 0.73 (0.45−1.18) | 0.202 |  | 0.70 (0.52−0.94) | 0.020 |
| **Age in years** |  |  |  |  |  |
| Age group ≤15 | 1.00 |  |  | 1.00 |  |
| Age group ≥ 35 | 1.12 (0.67−1.86) | 0.670 |  | 0.86 (0.63−1.17) | 0.345 |
| **Education** |  |  |  |  |  |
| Illiterate or primary | 1.00 |  |  | 1.00 |  |
| Secondary and above | 1.33 (0.69−2.53) | 0.391 |  | 0.92 (0.59−1.44) | 0.725 |
| **Profession** |  |  |  |  |  |
| Student/Teacher | 1.00 |  |  |  |  |
| Farmer/Fisherman | 1.17 (0.70−1.94) | 0.553 |  | 0.89 (0.65−1.20) | 0.440 |
| **Parasitic infections** |  |  |  |  |  |
| ***Schistosoma mekongi*** |  |  |  |  |  |
| Negative | 1.00 |  |  | 1.00 |  |
| Positive | 1 (omitted) |  |  | 3.87 (0.24−62.26) | 0.338 |

c*OR*: Crude odds ratio; *CI*: Confidence interval

| **Additional file 1: (continues)** | | | | | |
| --- | --- | --- | --- | --- | --- |
| **Indicators** | **Trace as negative** | | **Trace as positive** | | |
|  | **cOR (95% CI)** | ***P-*value** |  | **cOR (95% CI)** | ***P-*value** |
| ***O. viverrini*** |  |  |  |  |  |
| Negative | 1.00 |  |  | 1.00 |  |
| Positive | 1.59 (0.97−2.59) | 0.063 |  | 1.65 (1.22−2.23) | 0.001 |
| **Hookworm** |  |  |  |  |  |
| Negative | 1.00 |  |  | 1.00 |  |
| Positive | 0.59 (0.37−0.96) | 0.033 |  | 0.59 (0.44−0.79) | 0.001 |
| ***T. trichiura*** |  |  |  |  |  |
| Negative | 1.00 |  |  | 1.00 |  |
| Positive | 0.45 (0.16−1.25) | 0.126 |  | 0.51 (0.29−0.90) | 0.021 |
| ***A. lumbricoides*** |  |  |  |  |  |
| Negative | 1.00 |  |  | 1.00 |  |
| Positive | 0.93 (0.28−3.07) | 0.906 |  | 0.34 (0.12−0.95) | 0.041 |
| **Urine analysis** |  |  |  |  |  |
| **Leukocyte and/or Nitrite** |  |  |  |  |  |
| Negative | 1.00 |  |  | 1.00 |  |
| Positive | 1.78 (1.10−2.89) | 0.018 |  | 1.79 (1.32−2.42) | 0.001 |
| **Hematuria** |  |  |  |  |  |
| Negative | 1.00 |  |  | 1.00 |  |
| Positive | 1.67 (1.01−2.76) | 0.046 |  | 1.62 (1.17−2.23) | 0.003 |
| **Proteinuria** |  |  |  |  |  |
| Negative | 1.00 |  |  | 1.00 |  |
| Positive | 1.57 (0.84−2.94) | 0.160 |  | 1.45 (0.96−2.19) | 0.077 |
| **Pregnancy test** |  |  |  |  |  |
| Negative | 1.00 |  |  | 1.00 |  |
| Positive | 2.01 (0.24−16.59) | 0.515 |  | 1.29 (0.26−6.44) | 0.755 |
| c*OR*: Crude odds ratio; *CI*: Confidence interval | | | | | |
